# Antithrombotic Dilemmas after Left Atrial Appendage Occlusion Watchman Device Placement

**DOI:** 10.1155/2019/5247105

**Published:** 2019-04-28

**Authors:** Tania Ahuja, Scarlett Murphy, Daniel J. Sartori

**Affiliations:** ^1^New York University Langone Health, Department of Pharmacy, 550 First Avenue, New York, NY 10016, USA; ^2^New York University Langone Health, Department of Medicine, 550 First Avenue, New York, NY 10016, USA

## Abstract

Antithrombotic therapy for stroke prevention in patients with atrial fibrillation (AF) has dramatically shifted from warfarin, a vitamin K antagonist, to the direct oral anticoagulants (DOACs) such as dabigatran, apixaban, and rivaroxaban. In patients with contraindications to oral anticoagulation, left atrial appendage occlusion (LAAO) devices, such as the Watchman™ device, may be considered; however, temporary postimplantation antithrombotic therapy is still a recommended practice. We present a case of complex antithrombotic management, post LAAO device implantation, designed to avoid drug interactions with concomitant rifampin use and remained necessary secondary to subsequent device leak. This case highlights the challenges of antithrombotic therapy post LAAO device placement in a complex, but representative, patient.

## 1. Introduction

Antithrombotic therapy for stroke prevention in patients with atrial fibrillation (AF) has dramatically shifted from warfarin, a vitamin K antagonist, to the direct oral anticoagulants (DOACs) such as dabigatran, apixaban, and rivaroxaban [1–4]. In patients with contraindications to oral anticoagulation, left atrial appendage occlusion (LAAO) devices, such as the Watchman™ device (Boston Scientific Corporation; Natick, MA), may be considered [5, 6]. After device implantation, prevention of device-related thrombosis is essential during endothelialization. Prospective studies that investigated the Watchman™ device utilized antithrombotic strategies consisting of warfarin for 45 days post placement, followed by dual antiplatelet therapy (DAPT) for 6 months. However, DOACs, in conjunction with aspirin for 3 months, constitute an alternative regimen post device placement [7–10]. Indeed, real-life registry data reveal a spectrum of varied antithrombotic therapy currently in use, which is likely informed by a patient history of major bleeding or ineligibility for oral anticoagulation therapy [9]. The use of alternative anticoagulants, such as low-molecular-weight heparin (LMWH), post LAAO devices remains unknown. We present a case of complex antithrombotic management post Watchman™ device implantation designed to avoid drug interactions with concomitant rifampin use and subsequent device leak.

## 2. Case Report

The patient is a 70-year-old man, with a history of nonvalvular atrial fibrillation (NVAF) with prior stroke, chronic obstructive lung disease, hypertension, diabetes mellitus, chronic foot ulcers, and frequent falls, who initially presented with a fall 5 days after Watchman™ device placement. His history of NVAF was complicated by multiple episodes of syncope, despite various strategies including antiarrhythmic therapy. He was previously on dabigatran which resulted in severe bruising and rivaroxaban which was intolerable due to headaches. After discussion with his cardiologist, a Watchman™ device was placed given his high thrombotic risk and prior complications with oral antithrombotic therapy (CHADS2-VASC2 score of 6 and HAS-BLED score of 4) [11, 12]. After device placement, he was started on apixaban 5 mg twice daily plus aspirin 81 mg daily, with the plan for continuation for the following 45 days. However, 5 days after device placement, he presented to the emergency room after a fall, with lethargy, fever, and hypotension. He was found to be in septic shock from methicillin-resistant *staphylococcus aureus* (MRSA) bacteremia. His hemodynamics and mental status initially improved in the intensive care unit (ICU) on vasopressors, stress dose steroids, and broad-spectrum antibiotics, which were subsequently narrowed to intravenous vancomycin. In addition, his apixaban was transitioned to intravenous unfractionated heparin upon presentation to the ICU. In spite of hemodynamic improvement, his mental status worsened. Magnetic resonance imaging (MRI) of the brain revealed multiple acute small punctuate infarcts in the left corona radiata, right occipital cortex, and right frontal deep white matter. These multifocal strokes were thought to be cardioembolic, not septic in origin. Upon further questioning, the patient admitted to missing doses of apixaban post LAOO device placement. A transesophageal echocardiogram (TEE) was obtained; no thrombus in the left atrial appendage was noted, and there were no vegetations or patent foramen ovale. A well-positioned 27 mm Watchman™ device was visualized occluding the LAA orifice with a 1.3 mm paradevice leak, stable from prior. Given high concern for potential seeding of the newly implanted Watchman™ device, a six-week course of intravenous vancomycin plus oral rifampin was recommended by infectious disease consultants as a means of targeting MRSA and additionally preventing biofilm formation and device seeding. This was to be followed by a prolonged suppressive course of oral antibiotics.

The patient improved clinically with recovery of mental status and was deemed ready for transition from unfractionated heparin to an oral anticoagulant. Given the concerns about significant drug interactions with either warfarin or a DOAC with concomitant rifampin, a strong cytochrome (CYP) 3A4 inducer, strong permeability glycoprotein (Pgp) inducer, and moderate CYP2C9 inducer, a shared decision was reached to use weight-based LMWH with enoxaparin to complete the 45 days post Watchman™ device implantation.

The patient refused repeat TEE at 45 days, and anticoagulation therapy was continued with enoxaparin until the device could be evaluated. Two months after Watchman™ device placement, a repeat TEE was performed, revealing improvement in the paradevice leak from 1.3 mm to 1 mm, and the antithrombotic therapy was transitioned to DAPT with aspirin 81 mg daily and clopidogrel 75 mg daily. A repeat brain MRI obtained 4 months post LAOO device placement noted a right cerebellar chronic hemorrhage with numerous susceptibility foci distributed in the bilateral supratentorial and infratentorial compartment predominantly in the periphery of the brain parenchyma. Given the short interval since the prior MRI, the presence of these foci was concerning for microemboli of likely cardiac origin. A repeat TEE did not reveal a thrombogenic focus; however, there was persistence of paradevice leak at 1.3 mm. Anticoagulation therapy with apixaban was resumed, and DAPT was discontinued, due to the persistent device leak. The patient continues to struggle with worsening frequent falls; however, he remains functional at home with continued rehabilitation with physical therapy. He continues to take apixaban, living with the risks of associated bleeding that Watchman™ device placement was intended to reduce.

## 3. Discussion

The Watchman™ device, a LAAO device, has become an established alternative to oral anticoagulation in patients at risk for stroke with AF who have contraindications to anticoagulant therapy. Prevention of acute device-related thrombosis is critical following initial device placement, which paradoxically requires anticoagulant use. Although strategies vary, warfarin or a DOAC may be considered postimplant [7–9]. Low molecular weight heparins such as enoxaparin offer an attractive alternative to DOACs if oral anticoagulants are contraindicated. However, LMWHs have not been prospectively evaluated for the prevention of device related thrombosis. DAPT monotherapy constitutes a permissible alternative to oral anticoagulation, as real-world registry data reflects that patients with a history of major bleeding or other hard contraindications to anticoagulation therapy have received DAPT exclusively postimplant without an increase in device-related thrombosis [9].

In the case, rifampin was recommended as an adjunct to intravenous vancomycin in hopes of preventing biofilm formation of the Watchman™ device in the setting of known MRSA bacteremia, complicating anticoagulant selection. Due to the concerns of potential significant drug interaction between rifampin and the DOACs, these agents were initially deferred. Warfarin, a vitamin K antagonist, can be dose adjusted to overcome the induction from rifampin; however, there remains a risk of warfarin resistance, making this option challenging as well [13, 14]. This patient's course was further complicated by leakage into the left atrial appendage due to incomplete closure with the Watchman™. This complication has been noted in up to one-third of patients after placement [15]. Despite pervasive concerns about thrombosis, incomplete closure with the device may not lead to increase in thromboembolic events, according to a subanalysis of PROTECT AF [16]. However, given the gravity of possible stroke and systemic embolism with paradevice leaks, many clinicians prefer to continue anticoagulation therapy, as was seen in our case. Given the patient's high thrombotic risk (CHADS_2_-VASC_2_ of 6 and concomitant paradevice leak), a strategy of parenteral subcutaneous anticoagulation with enoxaparin was chosen for the postimplant period. He was later transitioned to DAPT, but as the leak reoccurred and the MRI revealed new foci, DAPT was replaced with a strategy of oral anticoagulation with apixaban selected as the patient was no longer receiving rifampin.

This case highlights the challenges with antithrombotic therapy post Watchman™ device placement, due to drug interactions and concern for stroke and systemic embolic due to device leak. A patient should carefully be selected for consideration for Watchman™ device placement, as anticoagulation therapy is often necessary at least temporarily after implantation and decision making regarding choice of anticoagulation may not always be able to be supported by the existing body of literature.
